# Assessment of measurement properties of peak VO_2_ in children with pulmonary arterial hypertension

**DOI:** 10.1186/1471-2466-12-54

**Published:** 2012-09-10

**Authors:** Joseph C Cappelleri, Lie-Ju Hwang, Jack Mardekian, Marko A Mychaskiw

**Affiliations:** 1 Department of Statistics, Pfizer Inc, Groton, CT, USA; 2 Department of Statistics, Specialty Care Business Unit, Pfizer Inc, New York, NY, USA; 3 Department of Outcomes Research, Specialty Care Business Unit, Pfizer Inc, Collegeville, PA, USA

## Abstract

**Background:**

The 6-minute walk test evaluates the effect of pharmacologic intervention in adults with pulmonary arterial hypertension (PAH) but, for reasons of compliance or reliability, may not be appropriate for children at all ages. Thus, peak oxygen consumption (VO_2_, maximal exercise test) was used instead in a pediatric PAH trial (STARTS-1) to evaluate pharmacologic intervention with sildenafil. This was the first large placebo-controlled trial to use the peak VO_2_ endpoint in this population. Our working hypothesis was that, as with other populations, percentage changes in peak VO_2_ in pediatric patients with PAH are reliable and are associated with changes in other clinical endpoints.

**Methods:**

Using data from the subpopulation of 106 patients who were developmentally and physically able to perform exercise testing, all of whom were World Health Organization Functional Class (WHO FC) I, II, or III, reliability was assessed using the intraclass correlation coefficient and Bland-Altman plot on screening and baseline data. Relationships between percentage change in peak VO_2_ from baseline to end of treatment and other endpoints were evaluated using correlation coefficients and regression analyses.

**Results:**

The intraclass correlation was 0.79 between screening and baseline peak VO_2_, an agreement that was supported by the Bland-Altman plot. Percentage change in peak VO_2_ correlated well (*r* ≥0.40) and showed responsiveness to a physician global assessment of change and with change in WHO FC (for baseline classes I and III). Percentage change in peak VO_2_ did not correlate with change in the Family Cohesion of the Child Health Questionnaire (*r* = 0.04) or with a subject global assessment of change (*r* = 0.12). The latter may have been influenced by child and parental-proxy response and instrument administration.

**Conclusion:**

In pediatric PAH patients who are developmentally and physically able to perform exercise testing, peak VO_2_ measurements exhibited good reliability and improvements that were associated with improvements in certain other clinical endpoints, such as the WHO FC and a physician global assessment.

**Trial registration:**

ClinicalTrials.gov identifier NCT00159913.

## Background

Pulmonary arterial hypertension (PAH) is a relatively rare condition associated with high mortality [1]. It is characterized by increased pulmonary vascular resistance and pulmonary arterial pressure leading to right ventricular failure and ultimately death [2]. It may be inherited (heritable PAH [HPAH], classified as familial or sporadic), develop spontaneously (idiopathic PAH [IPAH]), or occur in association with congenital heart defects, connective tissue disease, or other causes (associated PAH [APAH]) [3]. Oral sildenafil citrate (REVATIO®, Pfizer Inc, New York, NY) has been found to be efficacious and generally well tolerated in the treatment of chronic PAH in adults, both as disease-specific monotherapy and as add-on to intravenous therapy with epoprostenol [4,5]. However, safe and effective therapy to increase the functional capacity, quality of life, and survival of pediatric patients with PAH is also needed.

A widely used, noninvasive technique to assess PAH severity and response to treatment is the 6-minute walk test, which is based on improvements in submaximal exercise capacity [6,7]. However, when the first large, multicenter, randomized, double-blind, placebo-controlled clinical trial to investigate the effectiveness of sildenafil treatment for PAH in children who require treatment despite conventional therapy was being designed (ClinicalTrials.gov: NCT00159913) [8], many specialists believed that compliance with the directions for the 6-minute walk test could be difficult for children. Children may become uninterested or demotivated by factors unrelated to PAH, which could impact reliability of the test. Additionally, they may walk at a variable pace, resulting in unreliable or unstable measurements. Thus, for the design of the clinical trial, it was decided to use formal cardiopulmonary exercise testing that could be more readily standardized.

The ability to perform aerobic work is defined by peak oxygen consumption (VO_2_) at maximal effort [9]. Peak VO_2_ is a parameter of noninvasive cardiopulmonary exercise testing that is affected by age, sex, conditioning status, disease, or medications. Its prognostic value in terms of survival has been demonstrated in adult patients with IPAH [10]. Thus, percentage change from baseline to end of treatment in peak VO_2_ was selected as the primary efficacy endpoint in the controlled clinical trial of sildenafil treatment for PAH in children, making it the first trial of its kind with the potential to evaluate the correlation between changes in peak VO_2_ and other clinical endpoints [8].

The aim of this paper is to investigate the measurement properties of peak VO_2_ in terms of its associations with other clinical endpoints and its reliability. It was hypothesized that, as observed with other populations, percentage changes in peak VO_2_ in pediatric patients with PAH are reliable and are associated with changes in certain clinical endpoints.

## Methods

### Data Set

The data set was derived from the **S**ildenafil in **T**reatment-naive children, **A**ged 1–17 years, with pulmona**r**y ar**t**erial hyperten**s**ion (STARTS-1) trial, a multinational trial of sildenafil citrate with a 16-week, double-blind, placebo-controlled treatment phase [8]. Pediatric patients (aged 1–17 years) weighing ≥8 kg were included if they had IPAH, HPAH, or APAH associated with congenital heart defects or connective tissue disease. PAH (defined as mean pulmonary artery pressure ≥25 mmHg at rest, pulmonary capillary wedge pressure ≤15 mmHg [or mean left atrial pressure ≤15 mmHg or left ventricular end-diastolic pressure ≤15 mmHg], and as pulmonary vascular resistance index ≥3 Wood units × m^2^) was confirmed by right heart catheterization at baseline. Concurrent medication remained stable throughout the trial except for changes made for safety reasons. Nitrates, cytochrome P450 3A4 inhibitors, prostacyclin analogues, endothelin receptor antagonists, phosphodiesterase type 5 inhibitors (other than study medication), and arginine supplements were not allowed.

The trial was conducted in compliance with the ethical principles of the Declaration of Helsinki. The final protocol, any amendments, and informed consent documentation were reviewed and approved by the Institutional Review Boards and/or Independent Ethics Committees at each of the investigational centers participating in the study.^a^ Written informed consent was obtained from each child’s legal guardian and assent from each child when applicable.

Patients were stratified by developmental ability to perform cardiopulmonary exercise testing (bicycle ergometer) and by weight. Dosage of sildenafil was dependent on weight and doses were selected to achieve maximum plasma concentrations of 47 (low dose), 140 (medium dose), and 373 (high dose) ng/mL at steady state [8]. The 8-kg to 20-kg group was randomized 1:2:1 to sildenafil medium (10 mg) and high (20 mg) doses and placebo, respectively. The >20-kg to 45-kg group was randomized 1:1:1:1 to sildenafil low (10 mg), medium (20 mg), and high (40 mg) doses and placebo, respectively. The >45-kg group was randomized 1:1:1:1 to sildenafil low (10 mg), medium (40 mg), and high (80 mg) doses and placebo, respectively. Study medication was administered 3 times daily, ≥6 hours apart for 16 weeks. All patients randomized to sildenafil received 10 mg 3 times daily for 1 week followed by titration to assigned dose. A total of 234 patients were randomized and treated, of whom 115 were developmentally and physically able to perform exercise testing.

The primary efficacy endpoint in the STARTS-1 trial was percentage change in peak VO_2_ (normalized to body weight), measured in mL/kg/min, from baseline to week 16 or end of treatment (at trough plasma concentrations [before dosing or ≥4 h postdose]). Peak VO_2_ was assessed by cardiopulmonary exercise testing in those who were developmentally able to participate and achieved functional capacity limits for peak VO_2_ of ≥10 mL/kg/min and ≤28 mL/kg/min at screening [8]. Other endpoints used in the current correlational analyses included the following: a physician global assessment of change (PGA) and a subject/parent global assessment of change (SGA), which are 7-point rating scales (“markedly improved,” “moderately improved,” “mild improvement,” “no change,” “slightly worse,” “moderately worse,” and “markedly worse”); World Health Organization Functional Class (WHO FC, in which FC I represents no limitation of physical activity, FC II represents slight limitation, FC III represents marked limitation, and FC IV represents inability to carry out any physical activity without symptoms) [11]; and the Family Cohesion domain of the parent form of the Child Health Questionnaire [12].

### Correlational analyses

The analysis plan was formed prospectively (before conducting any analysis), with all analyses conducted in SAS/STAT® Version 8.2 (SAS Institute, Cary, NC). Analyses were based on peak VO_2_ data collected at baseline and at the end of treatment.

#### Reliability

Reliability refers to the reproducibility of the measurement when repeated at random in the same patient. Patients whose peak VO_2_ status has not changed should have a similar, or repeatable, response each time they are assessed. If there is considerable variability, the measurements are unreliable and results will be uninterpretable.

To assess test-retest reliability (stability), we examined the strength of agreement between peak VO_2_ pretreatment measurements at screening and baseline (up to 21 d after screening); no post-randomization data were used. We calculated the intraclass correlation (ICC) along with its confidence interval (CI), which estimates the proportion of all variation that is not due to measurement error [13,14]; a value ≥0.7 indicates acceptable reliability [15]. We also calculated the Pearson correlation coefficient, which gauges the magnitude of the linear relationship between the screening and baseline measurements. In addition, we constructed a Bland-Altman plot, which depicts agreement between screening and baseline measurements [16].

#### Associations with Peak VO_2_

Associations were evaluated by calculating Pearson correlation coefficients between the percentage change (baseline to end of treatment) in peak VO_2_ and each of following measures: the PGA; the SGA; change (baseline to end of treatment) in WHO FC by baseline FC; and change (baseline to end of treatment) in the Family Cohesion domain [12]. In sensitivity analyses, the corresponding Spearman-rank correlation coefficients were also examined.

For each of the prespecified correlational analyses, three sets of Pearson correlations were calculated: (1) pooled across treatment groups, (2) by treatment group (placebo separate from all sildenafil groups combined), and (3) partial, adjusting for (or partialing out) treatment. Differences in results among them were noted. It was hypothesized that associations would be meaningful (≥0.40, consistent with a meaningful correlation [17]) between percentage change in peak VO_2_ and all of the other measures except for change in the Family Cohesion domain. Correlation coefficients less than 0.30 were taken as less than meaningful [18]. Those between 0.30 and 0.40 were taken as ambiguous in their import.

#### Responsiveness

Responsiveness of measurement, a type of correlational analysis, addresses the ability to detect change when a particular patient improves or deteriorates. We assessed this association by comparing percentage change (baseline to end of treatment) in peak VO_2_ with change (baseline to end of treatment) in the WHO FC (categorized by baseline FC), the PGA, and the SGA. A regression analysis was applied to examine each of those relationships, with percentage change in peak VO_2_ serving as the outcome or dependent variable and each of the other measures serving as a separate predictor or explanatory variable. In each bivariate analysis, a regression model was fit in two ways: with the predictor taken as a discrete categorical variable and as a continuous variable.

## Results

Of the 115 patients who were developmentally able to perform exercise testing, 63% were girls, 36% had IPAH/HPAH, and the remainder had PAH associated with congenital heart defects; no patient was in FC IV at baseline. The mean ± standard deviation age was 12.7 ± 2.6 years (range, 7–17 years, with 39 [46%] age 7–12 and 46 [54%] age 13–17) in the combined sildenafil groups (n = 85), and was 11.6 ± 2.5 years (range, 7–16 years, with 18 [60%] age 7–12 and 12 [40%] age 13–17) in the placebo group (n = 30; Table 1). From this subgroup, 9 lacked postbaseline data because of machine failure/damage (n = 3), discontinuation without final assessment (n = 2), and too ill, inadequate test data, lack of staff, and not done in error (n = 1 each). Thus, 106 patients were evaluable for peak VO_2_ responses and provided data for the psychometric analyses. WHO FC data were available for 104 evaluable patients at baseline (27 FC I, 56 FC II, 21 FC III).

**Table 1:** Demographic and baseline clinical characteristics of patients able to exercise reliably

	**Placebo (N = 30)**	**Sildenafil (N = 85)**
Female sex, n (%)	19 (63)	53 (62)
Age, y, n (%)		
5–12	18 (60)	39 (46)
13–17	12 (40)	46 (54)
Race, n (%)		
White	8 (27)	27 (32)
Black	2 (7)	1 (1)
Asian	4 (13)	20 (24)
Other	16 (53)	37 (44)
Weight, kg, mean (range)	37 (20–60)	40 (15–106)
BMI, kg/m^2^, mean (SD)	18 (3)	18 (4)
Peak VO_2_, mL/kg/min, mean (SD)	20.0 (3.7)	17.7 (4.2)
WHO functional class, n (%)		
I	10 (33)	19 (22)
II	17 (57)	44 (52)
III	3 (10)	20 (24)
Missing	0	2 (2)
Etiology, n (%)		
IPAH/HPAH	10 (33)	31 (36)
Surgical repair*	7 (23)	22 (26)
Congenital systemic-to-pulmonary shunt (SaO_2_ ≥88% at rest)	12 (40)	30 (35)
Post-repair D-transposition of great arteries	1 (3)	2 (2)
Mean pulmonary artery pressure, mmHg, mean (SD)	58.7 (19.9)	68.4 (23.1)
Cardiac index, L/min/m^2^, mean (SD)	3.4 (1.4)	3.0 (1.1)
Pulmonary vascular resistance index, dyn·s/cm^5^/m^2^, mean (SD)	1299 (775)	1871 (1232)
Mean pulmonary capillary wedge pressure, mmHg, mean (SD)	10.0 (3.6)	9.7 (4.5)
Mean right atrial pressure, mmHg, mean (SD)	8.3 (4.5)	8.4 (4.9)

BMI = body mass index; HPAH = heritable PAH; IPAH = idiopathic PAH; n = sample size; PAH = pulmonary arterial hypertension; SaO_2_ = systemic arterial oxygen saturation; SD = standard deviation; VO_2_ = oxygen consumption; WHO = World Health Organization.

*Surgical repairs included atrial septal defect, ventricular septal defect, patent ductus arteriosus, aortopulmonary window, and others.

### Reliability

**Figure 1:**
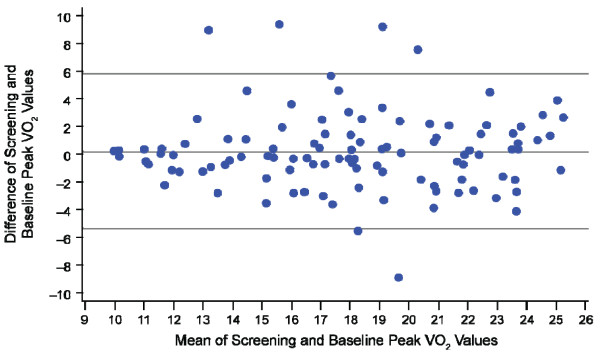
**Bland-Altman plot assessing the agreement between screening and baseline mean peak VO**_**2**_**.** Note: mean (standard deviation) difference = 0.23 (2.81). VO_2_ = oxygen consumptio7n.

The estimated ICC between screening and baseline peak VO_2_ was 0.79 (95% CI, 0.71–0.85; *P* < 0.0001), suggesting good reliability (>0.70). The same estimate of 0.79 was obtained from the Pearson correlation coefficient. Good agreement between the screening peak VO_2_ and baseline peak VO_2_ was also suggested by the Bland-Altman plot (Figure 1), which indicated no relationship between the difference in values across the 2 visits (which represents measurement error) and the mean of values across the 2 visits (which represents the true value). These data not only suggest that the data are reliable, but also support the use of the average of the two measurements as the baseline for statistical analyses.

### Associations

Across all treatment groups combined, Pearson correlations of percentage changes in peak VO_2_ from baseline with the PGA and with the change in WHO FC (with baseline FC of I and III) correlated well (correlations of ≥0.40; Table 2). Correlations of percentage changes in peak VO_2_ with the PGA depended on treatment group: correlation of 0.49 (95% CI, 0.30–0.64; *P* < 0.0001; n = 77) for sildenafil doses combined and −0.11 (95% CI, –0.46 to 0.27; *P* = 0.57; n = 29) for placebo. For patients with WHO FC II or I at baseline, there was little (FC II) or no (FC I) room for improvement in WHO FC. Thus, care needs to be taken with the interpretation of their data. Across all treatment groups combined, Pearson correlations of percentage changes in peak VO_2_ with changes in the Family Cohesion domain score and with the SGA were 0.04 and 0.12, respectively (Table 2) Results from Spearman correlations were very similar to those with Pearson correlations.

**Table 2:** **Correlation of percentage change (baseline to end of treatment) in peak VO**_**2**_**with other measures**

		**n**	**Pearson correlation (95% CI)**	***P *value**	**Spearman correlation (95% CI)**	***P *value**
Physician global assessment of change		106	0.41 (0.24 to 0.56)	<0.0001	0.40 (0.22 to 0.55)	<0.0001
WHO FC						
Baseline FC I*		27	0.40 (0.03 to 0.68)	0.04	0.41 (0.04 to 0.69)	0.03
Baseline FC II*		56	0.10 (−0.17 to 0.36)	0.45	0.03 (−0.24 to 0.29)	0.84
Baseline FC III		21	0.52 (0.11 to 0.78)	0.02	0.61 (0.24 to 0.82)	<0.01
Family Cohesion domain		83	0.04 (−0.18 to 0.25)	0.71	0.06 (−0.16 to 0.27)	0.57
Subject global assessment of change		104	0.12 (−0.07 to 0.31)	0.21	0.13 (−0.06 to 0.31)	0.19

CI = confidence interval; Family Cohesion domain = Family Cohesion domain of the Child Health Questionnaire [12]; n = sample size; VO_2_ = oxygen consumption; WHO FC = World Health Organization Functional Class.

*For patients with WHO FC II or I at baseline, there was little (FC II) or no (FC I) room for improvement in WHO FC. Thus, care needs to be taken with the interpretation of their data. For those with WHO FC I at baseline, only 4 patients deteriorated from baseline; for those with WHO FC II at baseline, none deteriorated and 8 improved from baseline.

### Responsiveness

**Figure 2:**
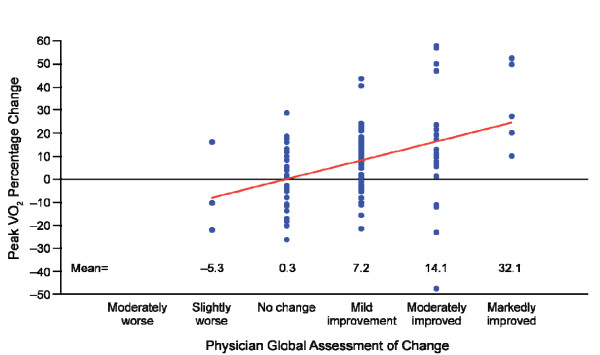
**Relationship of percentage change in peak VO**_**2**_**with the physician global assessment of change.** Percentage change in peak VO_2_ was for baseline to end of treatment values. Physician global assessment of change was responded to at the end of treatment. Note: the linear slope estimate, indicated by the straight line, was 8.0. A sensitivity analysis, which was performed excluding outliers, achieved similar results. VO_2_ = oxygen consumption.

**Figure 3:**
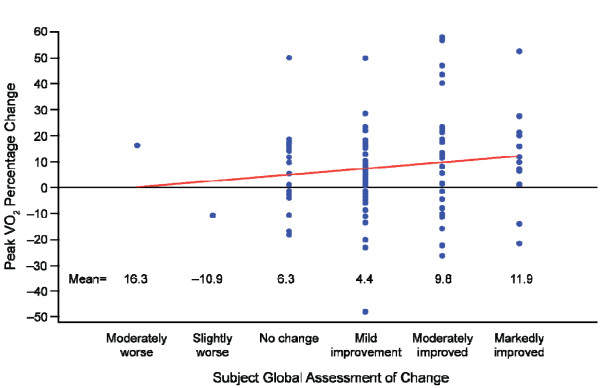
**Relationship of percentage change in peak VO**_**2**_**with the subject global assessment of change.** Percentage change in peak VO_2_ was for baseline to end of treatment values. Subject global assessment of change was responded to at the end of treatment. Note: the linear slope estimate, indicated by the straight line, was 2.2. A sensitivity analysis, which was performed excluding outliers, achieved similar results. VO_2_ = oxygen consumption.

For each categorical improvement on the PGA, the mean percentage change in peak VO_2_ increased by 8% (assuming a linear relationship, with the PGA taken as continuous; 95% CI, 4.6%–11.5%; *P* < 0.0001; Figure 2). For each categorical improvement on the SGA, the mean percentage change in peak VO_2_ increased by 2.2% (assuming a linear relationship, with the SGA taken as continuous; 95% CI, –1.3% to 5.8%; *P* = 0.21; Figure 3). The mean percentage changes in peak VO_2_, which were larger for higher improvement categories, were statistically significant for each of the three improvement categories of the PGA (7.2, 14.1, and 32.1; *P* < 0.01 for each) and in the two highest improvement categories of the SGA (9.8 and 11.9; *P* < 0.02 for each).

**Figure 4:**
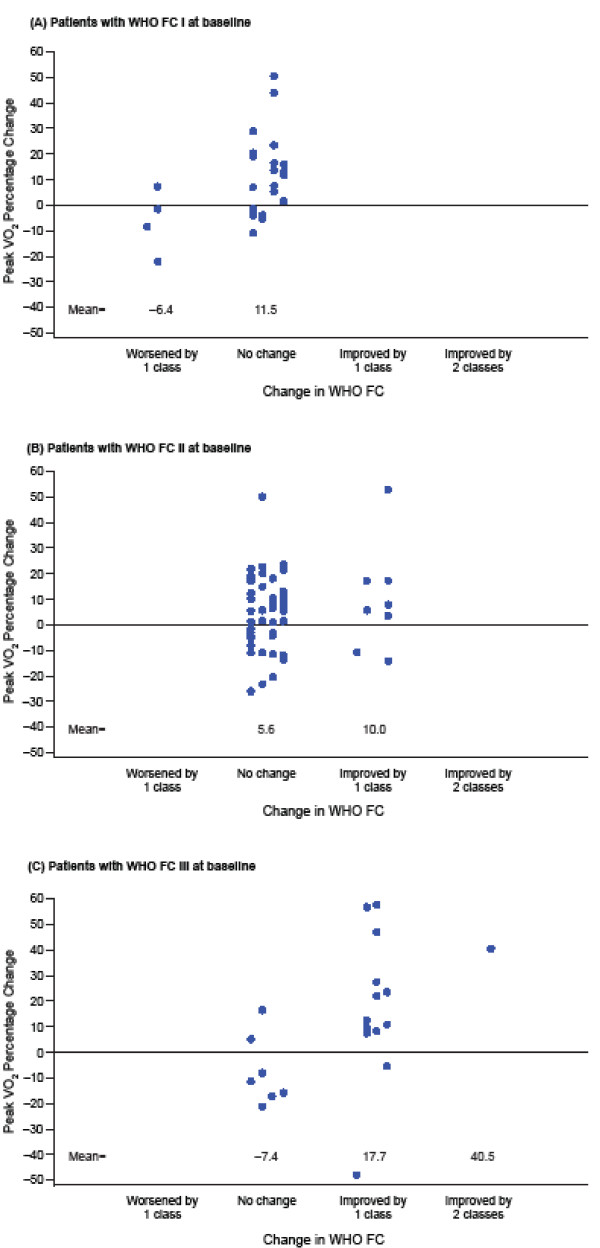
**Relationship of percentage change in peak VO**_**2**_**with change in WHO FC.** Percentage change in peak VO_2_ and change in WHO FC was for baseline to end of treatment values. Patients with WHO FC I (**A**), WHO FC II (**B**), and WHO FC III (**C**) at baseline. Note: no improvement was possible for patients with WHO FC I at baseline and improvement of only 1 FC was possible for patients with WHO FC II at baseline. VO_2_ = oxygen consumption; WHO FC = World Health Organization Functional Class.

For the 27 patients with WHO FC I at baseline, there was no room for improvement in FC: 3 of the 4 who deteriorated displayed a reduction in peak VO_2_ (Figure 4A). None of the 56 patients with WHO FC II at baseline deteriorated and only 8 improved; 6 of the 8 had an increase in peak VO_2_. Among all patients with WHO FC II at baseline, mean percentage change in peak VO_2_ increased by 4.40% (95% CI, –7.3% to 16.1%; *P* = 0.45; Figure 4B). None of the 21 patients with WHO FC III at baseline had a deterioration in WHO FC and 14 had an improvement, of whom 12 had an increase in peak VO_2_. Among all patients with WHO FC III at baseline, mean percentage change in peak VO_2_ increased by 24.6% (95% CI, 5.1%–44.2%; *P* = 0.02) for a 1-category improvement in WHO FC (Figure 4C).

## Discussion

In general, the results indicate that the peak VO_2_ has favorable measurement properties in pediatric patients with PAH who are developmentally and physically able to perform exercise testing. The magnitude of the correlation of mean percentage change in peak VO_2_ with the PGA was dependent on active or placebo treatment. This is to be expected because the placebo group is likely to have a more restricted range of values (which represent measurement variability and random fluctuations over time). In contrast, the active treatment group is likely to have a wider range of values (from the additional variability of individual treatment responses).

In a 16-week trial, it is not surprising that only 4 patients (all WHO FC I at baseline) reported deterioration in WHO FC. The importance of this endpoint is in the observance of improvement in WHO FC. However, for the large proportion of patients who were WHO FC I or II at baseline, there was no or limited room for improvement (unlike in WHO FC III patients). Eight of the 56 patients (14%) who were WHO FC II at baseline improved, but 14 of 21 patients (67%) who were WHO FC III at baseline improved. For these patients with WHO FC III at baseline, there was a strong positive association with percentage change in peak VO_2_.

It was unexpected that the percentage change in peak VO_2_ would share a low correlation with the SGA, and it may reflect influence by factors associated with child and parental-proxy responses and with instrument administration. A placebo response may have been observed with the SGA, in which patients (regardless of treatment group) are shifted toward a “mild improvement” response whether or not peak VO_2_ improves. In contrast, “markedly improved” on the SGA is unlikely to be caused by a placebo response and most such patients had clear improvement in peak VO_2_. This disparity can impair the correlation. The low correlation between the percentage change in peak VO_2_ and the SGA becomes less surprising given that a post-hoc correlation between PGA and SGA was not very high (0.39). The PGA correlated well with the change in WHO FC in the subgroup with baseline FC III but the SGA did not. The SGA is a mixture of parent and patient (child) responses, the meaning of which may be confounded, especially when the patient is young.

## Conclusions

This pediatric PAH trial—the largest one to date—offered the opportunity to evaluate peak VO_2_ as an endpoint with regard to its correlation with other clinical endpoints, such as the WHO FC and the PGA. Peak VO_2_ exhibited good reliability, and improvements were associated with improvements in certain other clinical endpoints. Additional research should be conducted to further elucidate the relationship between peak VO_2_ and the SGA, to inform use of the SGA in this patient population. This initial assessment of the measurement properties of peak VO_2_ suggests it is a robust measure with utility as a primary endpoint in clinical trials for the evaluation of the effect of drug treatment in pediatric PAH.

## Endnotes

^a^Royal Children's Hospital Ethics in Human Research Committee, Royal Children's Hospital, Parkville, VIC AUSTRALIA; Comitê de Ética em Pesquisa do Instituto Dante Pazzanese de Cardiologia, São Paulo, BRAZIL; The Hospital for Sick Children Research Ethics Board, Toronto, ON, CANADA; Health Research Ethics Board, Biomedical Research, University of Alberta Walter Mackenzie Health Science Centre, Edmonton, AB, CANADA; Children's and Women's Health Centre of BC Research Review Committee, Vancouver, BC, CANADA; Clinical Research Ethics Board, Vancouver, BC, CANADA; Comité Ético Científico Pediátrico, Santiago, CHILE; Comité de Evaluación Etico Científico, Hospital Dr. Sótero del Río Servicio de Salud Metropolitano Sur Oriente, Santiago, CHILE; Comite de Etica en Investigacion - Hospital Santa Clara – Empresa Social del Estado, Bogota, Cundinamarca, COLOMBIA; Comite de Etica en Investigacion Clinica - Fundacion Cardio Infantil, Instituto de Cardiologia, Departmento de Investigaciones, Bogota, Cundinamarca, COLOMBIA; Comite de Etica de la Clinica Cardiovascular, Medellin, Antioquia, COLOMBIA; Consejo Nacional de Investigacion en Salud, CONIS, Ministerio de Salud, San Jose, COSTA RICA; UCIMED Comite Etico Cientifico de la Universidad de Ciencias Medicas, San Jose, COSTA RICA; Latin Ethics, Guatemala, GUATEMALA; Medical Research Council Ethics Committee for Clinical Pharmacology, Budapest, HUNGARY; Institutional Ethics Committee, CARE Foundation - CARE Hospital, Hyderabad, Andhra Pradesh, INDIA; Research and Ethics Committee, Amrita Institute of Medical Sciences & Research Centre, Kochi, Kerala, INDIA; Comitato Etico dell'azienda ospedaliera di Bologna – Policlinico S.Orsola-Malpighi, Bologna, ITALY; Toho University Omori Medical Center Institutional Review Board, Ohta-ku, Tokyo, JAPAN; Joint Penang Independent Ethics Committee, Clinical Research Center, Gleneagles Medical Center, Penang, MALAYSIA; Comité de Bioética, Instituto Nacional de Cardiologia "Dr. Ignacio Chavez", Mexico, DF, MEXICO; Komisja Bioetyczna przy Instytucie, Pomnik Centrum Zdrowia Dziecka, Warszawa, POLAND; Komisja Bioetyczna Slaskiego, Uniwersytetu Medycznego w Katowicach, Katowice, POLAND; Komisja Bioetyczna Uniwersytetu Jagiellonskiego, Krakow, POLAND; Ethics Committee at the Federal Service on Surveillance in Healthcare and Social Development, Moscow, RUSSIAN FEDERATION; The Ethics Committee under Federal Agency of Quality Control Medicines, Moscow, RUSSIAN FEDERATION; Regionala etikprovningsnamnden i Lund, Lund, SWEDEN; Joint Institutional Review Board, Taipei, TAIWAN; National Taiwan University Hospital Ethics Committee, Taipei, TAIWAN; Western Institutional Review Board, Olympia, WA, UNITED STATES; Children's Hospital of Wisconsin, Milwaukee, WI, UNITED STATES; Children's Research Institute, Human Subjects Research Committee/CHRF Administration, Columbus, OH, UNITED STATES; Stanford University Medical Center Institutional Review Board, Stanford, CA, UNITED STATES; Colorado Multiple Institutional Review Board, Aurora, CO, UNITED STATES; Children's Hospital Boston, Committee on Clinical Investigators, Boston, MA, UNITED STATES; Washington University Medical Center Institutional Review Board, Human Studies Committee, St. Louis, MO, UNITED STATES; University of Michigan Institutional Review Board – Medicine, University of Michigan Hospitals and Health Systems, Ann Arbor, MI, UNITED STATES; Children's Hospital Medical Center Institutional Review Board, Seattle, WA, UNITED STATES; Medical University of South Carolina, Office of Research Integrity, Charleston, SC, UNITED STATES; Vanderbilt University Institutional Review Board, Nashville, TN, UNITED STATES.

## Competing interests

Joseph C. Cappelleri, Lie-Ju Hwang, Jack Mardekian and Marko A. Mychaskiw are employees of Pfizer Inc (USA), the manufacturer of sildenafil citrate.

## Authors’ contributions

All authors participated in varying ways to the conception, design, analysis, or interpretation of results; and to the drafting of the manuscript or to its revision for important intellectual content. In addition, all authors read and approved the final manuscript.

## Pre-publication history

The pre-publication history for this paper can be accessed here:

http://www.biomedcentral.com/1471-2466/12/54/prepub
